# Research on the modified tri-frequency combination model for determining geopotential using China space station microwave links

**DOI:** 10.1016/j.isci.2026.115869

**Published:** 2026-04-24

**Authors:** Pengfei Zhang, Chenxiang Wang, Wei Xu

**Affiliations:** 1 Key Laboratory of Surveying and Mapping Science and Geospatial Information Technology of MNR, Beijing 100036, China; 2 Hubei Luojia Laboratory, Wuhan 430079, China; 3 School of Geodesy and Geomatics, Wuhan University, Wuhan 430079, China; 4 State Key Laboratory of Spatial Datum, Henan University, Zhengzhou 450046, China; 5 School of Geographic Information and Tourism, Chuzhou University, Chuzhou 239000, China

**Keywords:** Applied sciences, Engineering, Geotechnical engineering

## Abstract

Determining the gravity potential (GP) is a major task for geodesy. Advances in atomic clocks enable relativistic geodesy. China Space Station (CSS) carries an optical clock with stability ${2\times 10}^{-15}/\sqrt{\tau }$, but no previous frequency comparison model matches its accuracy. Previous tri-frequency models required fixed frequency ratios and ignored higher-order ionospheric effects. In this study, an improved tri-frequency combination model is proposed that extends to the c-4 terms of the GP and accounts for Doppler shifts, tropospheric and higher-order ionospheric effects, tidal effects, and the Shapiro delay. Crucially, it can be applied to any arbitrary combination of three frequencies. To validate the performance of the modified model, we use CSS tri-frequency microwave links for space-to-ground comparison and simulate GP determination at ground stations. With EGM2008 as the truth, the model achieved 0.7 m^2^/s^2^ accuracy, supporting centimeter-level unification of global height datums. The model provides theoretical support for future real-data processing.

## Highlights

• A modified tri-frequency combination model with geopotential c^−4^ terms is proposed
• Modified model with tidal and higher-order ionospheric effects enables cm-level geopotential
• The simulation results show the modified model can obtain the GP with an accuracy of 0.7 m^2^/s^2^

## Graphical abstract

**Figure:**
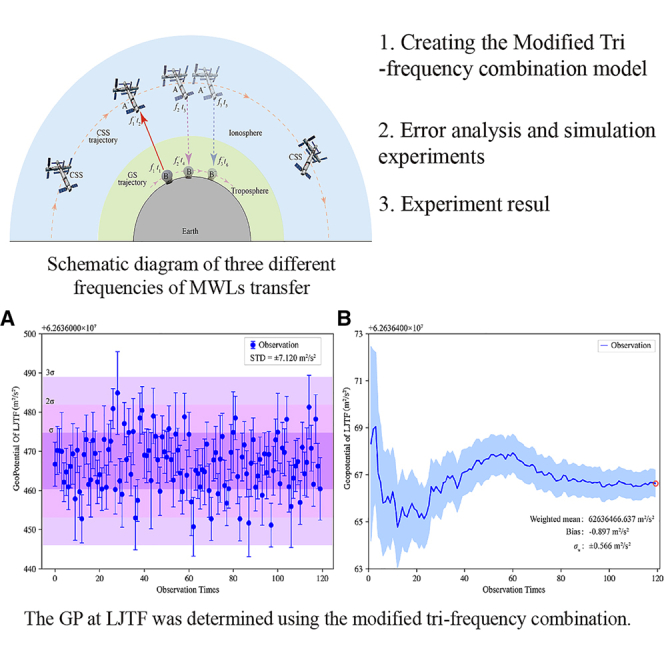

## Introduction

Determining the geopotential (GP) has long been a central objective in geodesy. However, the traditional approach of combining gravity measurements with spirit leveling is inefficient, and its accuracy diminishes as the survey line lengthens, making it difficult to achieve *trans*-oceanic or intercontinental measurements. This severely hinders the unification of the global height system. In recent years, with the continuous improvement in the accuracy of atomic clocks,^1^^,^^2^^,^^3^^,^^4^^,^^5^ methods for measuring geopotential differences (GPDs) based on general relativity theory (GRT) have advanced rapidly.^6^^,^^7^^,^^8^^,^^9^^,^^10^^,^^11^ According to the GRT, atomic clocks located at positions with different gravity potential (GP) values run at distinct frequencies. When the accuracy of a clock reaches 10^−18^, it can detect GPD approximately 0.1 m^2^/s2.^12^^,^^13^ This frequency shift enables the determination of GPD between two locations by comparing the time or frequency readings of the clocks.^14^^,^^15^^,^^16^

In recent years, by deploying high-precision optical atomic clocks (OACs) on spaceborne platforms and establishing space-to-ground comparison links, it has been possible to overcome issues such as the accumulation of measurement errors and low efficiency, which are difficult to solve by traditional GP measurement methods.^7^^,^^17^^,^^18^^,^^19^^,^^20^ Spaceborne atomic clocks are expected to play an increasingly vital role in areas such as time and frequency transmission, determination of GPD, and unification of height datums.^7^^,^^21^^,^^22^^,^^23^^,^^24^ The first experiment to test gravitational redshift (GRS), known as gravity probe A (GP-A), was conducted by the National Aeronautics and Space Administration (NASA) in 1976. The mission launched a rocket carrying an atomic clock to an altitude of 10,000 km. Using microwave frequency comparisons, the GRS was tested with an accuracy of 7 × 10^−5^.^25^^,^^26^ In 1996, the Atomic Clock Ensemble in Space (ACES) project was proposed through a collaboration between the European Space Agency (ESA) and the French Space Agency (CNES). The mission involved installing a high-precision atomic clock system on the exterior of the International Space Station (ISS).^27^ One of its key scientific goals was to apply relativistic geodesy techniques to determine the Earth’s GP with an accuracy equivalent to 10 cm in height and to reconstruct a wide-area gravity field model.^27^^,^^28^ The ACES payload includes a cesium atomic clock (PHARAO) and an active hydrogen maser (SHM), together providing frequency signals with stability on the magnitude of 10^–16^.^29^^,^^30^

China has also initiated its own spaceborne atomic clock program. On 22 October 2022, the Mengtian laboratory module was launched and docked with the China Space Station (CSS).^31^^,^^32^ After completing the docking, CSS will operate at an altitude of 350∼400 km with an inclination of 41.5° for more than 10 years.^32^^,^^33^^,^^34^^,^^35^ The Mengtian module carries a high-precision time and frequency system (HPTFS),^24^^,^^31^^,^^33^ which includes an OAC, a cold atomic microwave clock (CAMC), a hydrogen atomic clock (HAC), a femtosecond optical frequency comb (FOFC), and supporting equipment for time-frequency comparison.^24^^,^^31^^,^^33^ The frequency stabilities of the CAMC, HAC, and OAC are 2 × 10^−13^/ $\sqrt{\tau }$. 5 × 10^−14^/ $\sqrt{\tau }$, and 2 × 10^−15^/ $\sqrt{\tau }$, respectively, with long-term stabilities around 2 × 10^−15^ @ 1 day, 2 × 10^−16^ @ 1 day, and 3 × 10^−17^ @ 4000 s.^24^^,^^32^^,^^36^ The OAC onboard CSS is linked to the ground station (GS) OAC via laser link and microwave links (MWLs). The MWL system comprises two uplinks and two downlinks. The uplinks operate at 30.4 GHz and 26.8 GHz using left-hand circular polarization (LHCP) direction, while the downlinks use 30.4 GHz and 20.8 GHz with right-hand circular polarization (RHCP) direction.^16^^,^^24^^,^^32^

The HPTFS provides a critical foundation for high-precision space-to-ground time-frequency comparison. Numerous scholars have recently conducted research leveraging this system.^31^^,^^32^^,^^37^ For instance, Liu et al. (2018) improved the common-view (CV) time comparison method using the CSS, helping to overcome the blind-area limitation inherent in traditional approaches and achieving an accuracy of several tens of picoseconds.^36^ Guo et al. (2022) proposed a novel two-way time synchronization scheme, reaching space-to-ground synchronization accuracy better than 1.5 ps^31^ Shen et al. (2023) introduced a GRS test method based on dual-frequency transmission at the same frequency; simulation results indicated that the accuracy of this approach reaches the magnitude of 5 × 10^−7^.^38^ Additionally, Zhang et al. (2024) applied the dual-frequency combination method to determine the GP with an accuracy of 0.71 m^2^/s^2^. Sun et al. (2021) adapted the original model to be compatible with the ACES mission, taking into account the MWLs frequency comparison accuracy of 10^−16^. Simulation results indicate that the GRS test under this framework can achieve an accuracy of 4.91 × 10^−6^.^39^ However, the dual-frequency combination method must use two MWLs with the same frequency and different polarization directions. For the ACES tri-frequency combination model, the frequency ratios of the three MWLs must maintain a specific proportional relationship to eliminate first-order ionospheric delays. Additionally, the model does not account for higher-order ionospheric shifts and the c^−4^ term of GP; its accuracy is limited to levels less than 10^−18^. To overcome these limitations, this study leverages the characteristics of the HPTFS onboard the CSS to propose a modified tri-frequency combination model. The improved model incorporates the c^−4^ term of GP and higher-order ionospheric effects, achieving the precision required for 10^−18^ level microwave frequency transfer. It supports arbitrary tri-frequency combinations and offers a theoretical foundation for measuring the GPD between the CSS and GSs. This approach provides a novel method for unifying the global height datum with centimeter-level accuracy.

The rest of this paper is as follows: In Section 2, we describe the construction of a modified tri-frequency combination model, extending the model to c^−4^ terms, and correcting the errors for ionosphere, troposphere, and tidal influences. Section 3 outlines the methodology for the simulation experiments. We then analyze the experimental results in Section 4, with a focus on residuals and the accuracy of the GP values. Finally, we discuss the findings and present our conclusions in Section 5.

### Methods

#### One-way microwave frequency transfer model accurate to the c^−4^ terms

**Figure 1:**
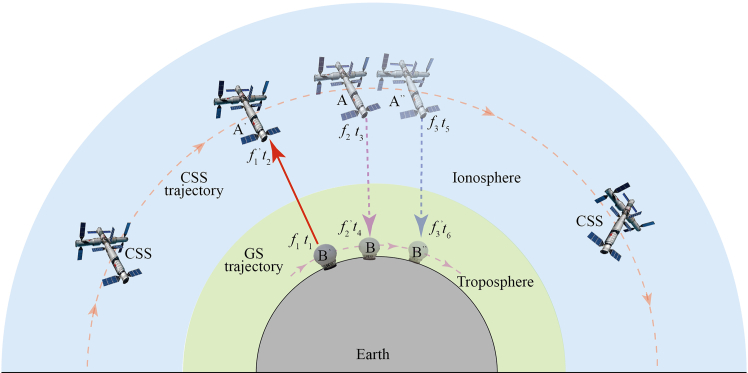
Schematic diagram of three different frequencies of MWLs transfer (Modified by Sun et al. 2021) At time *t*_1_, the GS emits an MWL with frequency *f*_1_ to the CSS, which receives it as $f_{1}^{\prime }$ at time *t*_2_. In response, the CSS emits two MWLs at frequencies *f*_2_ and *f*_3_ at time *t*_3_ and *t*_5_, which are received by the GS at times *t*_4_ and *t*_6_ as $f_{2}^{\prime }$ and $f_{3}^{\prime }$, respectively.

This study utilizes the geocentric celestial reference system (GCRS) as its coordinate frame. Within this system, the time coordinate is defined as *x*^0^/*c* = *t* (where *c* denotes the speed of light and *t* is the coordinate time), while the spatial coordinates *x* = (*x*^*i*^) are harmonic. Two points are considered: Point A, located on the CSS, with coordinates *x*_*A*_ = (*ct*_*A*_, *x*_*A*_), and Point B, a GS with coordinates *x*_*B*_ = (*ct*_*B*_, *x*_*B*_), as illustrated in Figure 1. A clock is associated with each point: clock $O_{A}$ at A with proper frequency *f*_A_, and clock $O_{B}$ at B with proper frequency *f*_B_. The GP difference between the two locations can be determined from the frequency ratio *f*_*A*_/*f*_*B*_. In practice, this ratio is obtained by transmitting MWLs from the CSS (A) to the GS (B)^12^^,^^40^:

(Equation 1) $$\frac{f_{A}}{f_{B}}=\left(\frac{f_{A}}{\nu_{A}}\right)\left(\frac{\nu_{A}}{\nu_{B}}\right)\left(\frac{\nu_{B}}{f_{B}}\right)$$

where *ν*_A_ denotes the proper frequency of the MWL measured at point A during the emission time *t*_*A*_ and *ν*_*B*_ represents the proper frequency observed at point B upon signal reception at time *t*_*B*_. The ratio *f*_*A*_/*ν*_*A*_ (first term) and *ν*_*B*_/*f*_*B*_ (third term) can be directly determined using local clocks $O_{A}$ and $O_{B}$ during signal emission and reception, respectively. During data processing, these measurements from *f*_*A*_/*ν*_*A*_ and *ν*_*B*_/*f*_*B*_ are incorporated into the MWL data in the form of clock errors.^24^ The primary challenge in one-way frequency transfer lies in accurately modeling the ratio *ν*_*A*_/*ν*_*B*_ or *ν*_*B*_/*ν*_*A*_, which is described by^12^:

(Equation 2) $$\frac{\nu_{A}}{\nu_{B}}=\frac{u_{A}^{\mu }{\left(l_{\mu }\right)}_{A}}{u_{B}^{\mu }{\left(l_{\mu }\right)}_{B}}$$

where $u_{A}^{\mu }$ and $u_{B}^{\mu }$ denote the unit 4-velocity of the clock $O_{A}$ and $O_{B}$, respectively, while ${\left(l_{\mu }\right)}_{A}$ and ${\left(l_{\mu }\right)}_{B}$ are the null tangent vectors at the emission point *x*_*A*_ and reception point *x*_*B*_, respectively.^12^

The coordinate velocities of clocks $O_{A}$ and $O_{B}$ are defined as *v*_*A*_=(*dx*/*dt*)_*A*_ and *v*_*B*_=(*dx*/*dt*)_*B*_, respectively. To enable the comparison of fractional frequency at the magnitude of 10^−18^, the free space frequency transfer model for *ν*_*A*_/*ν*_*B*_ or *ν*_*B*_/*ν*_*A*_ must be extended to order 1/c^−4^, and can be expressed as^12^^,^^40^:

(Equation 3) $$\frac{\nu_{B}}{\nu_{A}}=\frac{u_{B}^{0}}{u_{A}^{0}}\times \frac{q_{B}}{q_{A}},q_{A}=1+\frac{1}{c}l_{A}\cdot v_{A},q_{B}=1+\frac{1}{c}l_{B}\cdot v_{B}$$

where *l*_*A*_ and *l*_*B*_ are the quantities and can extend to the order 1/c^3^, following the definition given in.^12^ The ratio *q*_*B*_/*q*_*A*_ can be expanded as

(Equation 4) $$\begin{array}{l} \frac{q_{B}}{q_{A}}=1-\frac{1}{c}\frac{N_{AB}\cdot \left(v_{A}-v_{B}\right)}{1+N_{AB}\cdot \frac{v_{A}}{c}}+\frac{1}{c^{3}}\left[l_{B}^{\left(2\right)}\cdot v_{B}-l_{A}^{\left(2\right)}\cdot v_{A}\right]+\frac{1}{c^{4}}\left[l_{B}^{\left(3\right)}\cdot v_{B}-l_{A}^{\left(3\right)}\cdot v_{A}\right]-\frac{1}{c^{4}}N_{AB}\cdot [\left(l_{A}^{\left(2\right)}\cdot v_{A}\right)\\ \times \left(v_{B}-2v_{A}\right)+\left(l_{B}^{\left(2\right)}\cdot v_{B}\right)v_{A}]+O\left(-5\right) \end{array}$$

where *N*_*AB*_ = *R*_*AB*_/*R*_*AB*_,*R*_*AB*_ = *x*_*B*_-*x*_*A*_,*R*_*AB*_ = |*R*_*AB*_|. The terms are decomposed as $l_{A}^{\left(2\right)}/c^{2}=l_{M}\left(x_{A},x_{B}\right)+l_{J_{2}}\left(x_{A},x_{B}\right),l_{A}^{\left(3\right)}/c^{3}=l_{S}\left(x_{A},x_{B}\right)$ and similarly for point B: $l_{B}^{\left(2\right)}/c^{2}=l_{M}\left(x_{B},x_{A}\right)+l_{J_{2}}\left(x_{B},x_{A}\right),l_{B}^{\left(3\right)}/c^{3}=l_{S}\left(x_{B},x_{A}\right)$. Here *l*_*M*_, $l_{J_{2}}$, *l*_*S*_ are the contributions due to the Earth’s mass (primarily the Shapiro effect), quadrupole moment (J_2_) and the intrinsic angular momentum (S), respectively. In Equation 4, the second term on the right-hand side is the first-order Doppler shift, denoted as Δ*f*_dop_, with a magnitude of 10^−5^. The c^−3^ term is caused by M and J_2_, denoted as Δ*f*_shap_ and $\Delta f_{J_{2}}$, with the magnitudes of 10^−14^ and 10^−18^, respectively. The first c^−4^ term (denoted as Δ*f*_*S*_) is induced by intrinsic angular momentum S (in terms $l_{A}^{\left(3\right)}/c^{3}$ and $l_{B}^{\left(3\right)}/c^{3}$), while another c^−4^ term comes from $l_{A}^{\left(2\right)}/c^{2}$ and $l_{B}^{\left(2\right)}/c^{2}$, denotes as Δ*f*_*l*_. Both of c^−4^ terms are less than the magnitude of 2 × 10^−18^.

To maintain consistency with Equation 4, for a clock moving with the coordinate velocity *v*, the quantity 1/*u*^0^ is given by the formula^12^:

(Equation 5) $$\frac{1}{u^{0}}=1-\frac{1}{c^{2}}\left(V+\frac{1}{2}v^{2}\right)+\frac{1}{c^{4}}\left[\frac{1}{2}V^{2}-\frac{3}{2}Vv^{2}-\frac{1}{8}v^{4}+4V\cdot v\right]$$

where *V* is the vector gravitational potential, the value is *V*. The order of 1/c^4^ in *g*_00_ for $u_{B}^{0}/u_{A}^{0}$ can be expressed as:

(Equation 6) $$A_{\text{rel}}=\frac{u_{B}^{0}}{u_{A}^{0}}=\frac{1-\frac{1}{c^{2}}\left(V_{A}+\frac{1}{2}v_{A}^{2}\right)+\frac{1}{c^{4}}\left[\frac{1}{2}V_{A}^{2}-\frac{3}{2}V_{A}v_{A}^{2}-\frac{1}{8}v_{A}^{4}+4V_{A}\cdot v_{A}\right]}{1-\frac{1}{c^{2}}\left(V_{B}+\frac{1}{2}v_{B}^{2}\right)+\frac{1}{c^{4}}\left[\frac{1}{2}V_{B}^{2}-\frac{3}{2}V_{B}v_{B}^{2}-\frac{1}{8}v_{B}^{4}+4V_{B}\cdot v_{B}\right]}$$

The magnitude of c^−2^ and c^−4^ terms are approximately 10^−10^ and 10^−19^, respectively. From Equations 4 and 6, the frequency transfer model can be established

(Equation 7) $$\frac{\nu_{B}}{\nu_{A}}=A_{\text{rel}\;}\left(1+\Delta f_{\text{dop}\;}+\Delta f_{\text{shap}\;}+\Delta f_{J_{2}}+\Delta f_{S}+\Delta f_{l}\right)$$

Equation 7 describes frequency transfer in a vacuum. However, in practice, MWLs propagating through the atmosphere are subject to additional disturbances from the ionosphere and troposphere. The resulting Doppler frequency shift due to the phase path variation can be expressed as^41^^,^^42^:

(Equation 8) $$\frac{\Delta f}{f}=-\frac{1}{c}\frac{dP}{dt}$$

where c denotes the speed of light in the vacuum, f represents the proper frequency of the MWL, and P is the phase path. The refractive indices (*n*_*i*_,*n*_*t*_) and propagation paths (*L*_*i*_,*L*_*t*_) correspond to the ionosphere and troposphere. The frequency shifts due to atmospheric effects can then be expressed as^43^^,^^44^:

(Equation 9) $$\frac{\Delta f}{f}=\Delta f_{ion}+\Delta f_{tro}=-\frac{1}{c}\frac{d}{dt}\int_{L_{i}}\;\left(n_{i}-1\right)dl_{i}-\frac{1}{c}\frac{d}{dt}\int_{L_{t}}\;\left(n_{t}-1\right)dl_{t}$$

When considering the ionospheric refractive index *n*_*i*_, the resulting third-order ionospheric frequency shift, expressed as a fractional frequency shift, is given by^45^:

(Equation 10) $$\frac{{\Delta f}_{ion}=40.3\frac{1}{cf^{2}}\;\frac{\text{ds}}{dt}\bar{+}\frac{7527}{2f^{3}}\bar{B_{0}\;\cos\;\theta }\frac{ds}{dt}+812\cdot \frac{3}{\left(cf^{4}\right)\eta N_{m}}ds}{\text{dt}}$$

where ds/d*t* is the rate of change of slant total electron content (STEC), $\bar{B_{0}\;\cos\theta }$ denotes the average value for the magnetic field component *B*_0_*cos*⁡*θ*,^46^ and *η* is the shape parameter defined as $\eta =\int_{Li}\;N_{e}^{2}dl/\left(N_{m}*STEC\right)$.^47^ The positive sign (+) and negative sign (−) correspond to RHCP and LHCP MWLs, respectively. Equation 10 shows that the ionospheric frequency shift depends on the MWL frequency, and the second-order term is further influenced by the signal polarization and the magnetic field. By estimating the ionospheric frequency shifts in the CSS mission, the total ionospheric shift is found to be on the order of 10^−15^∼10^−13^.

Substituting the tropospheric refraction coefficient *n*_*t*_ into Equation 9 yields the tropospheric frequency shift^39^^,^^44^:

(Equation 11) $$\Delta f_{tro}=-\frac{1}{c}\frac{d}{dt}\int_{\text{Lt}}\;\left(M_{1}+M_{2}\right)dl_{t}$$

where *M*_1_ = 77.610^−6^*p*/*T*, and *M*_2_ = 0.373*κ*/*T*^2^, with temperature *T*, total pressure *p,* and partial pressure of water vapor *κ* along the trajectory. Equation 11 indicates that the tropospheric frequency shift is independent of the MWLs’ frequency. The magnitude of tropospheric frequency shifts ranges from 10^−13^ to 10^−10^.

In addition to the Earth’s geopotential influence, the perturbing tidal effects caused by external celestial bodies (including the Sun, Moon, and planets) must be incorporated into a precise model. The primary tidal gravitational effects are composed of the solid Earth tides, ocean tides, solid Earth pole tides, and ocean pole tides. Solid Earth tides represent the dominant contribution, generating daily vertical displacements of 20∼50 cm through the combined gravitational potential influence of the Sun and Moon.^48^ The tidal potential Δ*V*(*r*,*ϕ*,*λ*) at any geocentric position (*r*,*ϕ*,*λ*) is most rigorously expressed through a spherical harmonic expansion truncated at degree N, as follows^48^:

(Equation 12) $$\Delta V\left(r,\phi ,\lambda \right)=\frac{GM}{r}\sum_{n=0}^{N}\;{\left(\frac{R_{e}}{r}\right)}^{n}\times \sum_{m=0}^{n}\;\left[\Delta {\bar{C}}_{nm}\;\cos\left(m\lambda \right)+\Delta {\bar{S}}_{nm}\;\sin\left(m\lambda \right)\right]{\bar{P}}_{nm}\left(\sin\phi \right)$$

where *r* is the geocentric distance to the field point, *ϕ* and *λ* denote the geodetic latitude and longitude, *GM* and *R*_*e*_ represent the gravitational parameter and the equatorial radius of the Earth, respectively. In consideration of tidal effects, the geopotential V in Equation 6 can be replaced by *U*=*V*+Δ*V*_tide_. Consequently, Equation 6 can be rewritten as:

(Equation 13) $${\bar{A}}_{\text{rel}}=\frac{u_{B}^{0}}{u_{A}^{0}}=\frac{1-\frac{1}{c^{2}}\left(U_{A}+\frac{1}{2}v_{A}^{2}\right)+\frac{1}{c^{4}}\left[\frac{1}{2}U_{A}^{2}-\frac{3}{2}U_{A}v_{A}^{2}-\frac{1}{8}v_{A}^{4}+4U_{A}\cdot v_{A}\right]}{1-\frac{1}{c^{2}}\left(U_{B}+\frac{1}{2}v_{B}^{2}\right)+\frac{1}{c^{4}}\left[\frac{1}{2}U_{B}^{2}-\frac{3}{2}U_{B}v_{B}^{2}-\frac{1}{8}v_{B}^{4}+4U_{B}\cdot v_{B}\right]}$$

Since the c^−4^ terms in Equation 13 contribute at a level below 10^−18^, we consolidate them into a single correction term *ϵ*(*c*^−4^), leading to the simplified form:

(Equation 14) $${\bar{A}}_{\text{rel}}=\frac{u_{B}^{0}}{u_{A}^{0}}=1-\frac{1}{c^{2}}\left(U_{A}+\frac{1}{2}v_{A}^{2}-U_{B}-\frac{1}{2}v_{B}^{2}\right)+\epsilon \left(c^{-4}\right)$$

Where $\epsilon \left(c^{-4}\right)=\frac{1}{c^{4}}\left[\frac{1}{2}U_{A}^{2}-\frac{3}{2}U_{A}v_{A}^{2}-\frac{1}{8}v_{A}^{4}+4U_{A}\cdot v_{A}\right]-\frac{1}{c^{4}}\left[\frac{1}{2}U_{B}^{2}-\frac{3}{2}U_{B}v_{B}^{2}-\frac{1}{8}v_{B}^{4}+4U_{B}\cdot v_{B}\right]\text{.}$

By combining Equations 7, 10, 11, and 14, we derive the one-way frequency transfer model:

(Equation 15) $$\frac{\nu_{B}}{\nu_{A}}={\bar{A}}_{\text{rel}\;}\left(1+\Delta f_{\text{dop}\;}+\Delta f_{\text{shap}\;}+\Delta f_{J_{2}}+\Delta f_{S}+\Delta f_{l}+\Delta f_{\text{ion}\;}+\Delta f_{\text{tro}\;}\right)$$

#### Modified tri-frequency transfer combination model

The analysis in Section 2.1 indicates that the first-order Doppler shift, at a magnitude of 10^−5^, is the dominant error source in one-way microwave frequency transfer. Due to the limited orbit determination accuracy of the CSS, the first-order of the Doppler shifts can only be eliminated to about 10^−10^ for one-way frequency transfer. To address this limitation, we introduce a modified tri-frequency combination model designed to achieve higher transfer precision.

The following description details the model, which uses the six timestamps (*t*_1_∼*t*_6_) in Figure 1 to establish the tri-frequency combination. The sequence begins with the GS transmitting a frequency *f*_1_ at *t*_1_, which is received by the CSS as $f_{1}^{\prime }$ at *t*_2_. In response, the CSS transmits signals at frequencies *f*_2_ and *f*_3_ at times *t*_3_ and *t*_5_. The corresponding received signals at the GS at times *t*_4_ and *t*_6_ are denoted $f_{2}^{\prime }$ and $f_{3}^{\prime }$. The time intervals are defined as *T*_*ij*_ = *t*_*j*_-*t*_*i*_. While ideal synchronization implies *T*_23_ = *T*_35_ = 0, practical hardware delays mean these values are non-zero.

As analyzed in Section 2.1, the ionospheric frequency shift is the only error source that depends on the MWL frequency; frequency shifts from other sources are frequency *f*_2_ independent. The ratio is obtained by dividing the received-to-transmitted ratio for $\frac{f_{2}^{\prime }}{f_{2}}$ by that for $\frac{f_{3}^{\prime }}{f_{3}}$, yielding:

(Equation 16) $$\frac{f_{2}^{\prime }}{f_{2}}/\frac{f_{3}^{\prime }}{f_{3}}=\frac{{\bar{A}}_{\text{rel}}^{\left(2\right)}}{{\bar{A}}_{\text{rel}}^{\left(3\right)}}\times \frac{\left(1+\Delta f_{\text{dop}}^{\left(2\right)}+\Delta f_{\text{shap}}^{\left(2\right)}+\Delta f_{J_{2}}^{\left(2\right)}+\Delta f_{S}^{\left(2\right)}+\Delta f_{1}^{\left(2\right)}+\Delta f_{\text{ion}}^{\left(2\right)}+\Delta f_{\text{tro}}^{\left(2\right)}\right)}{\left(1+\Delta f_{\text{dop}}^{\left(3\right)}+\Delta f_{\text{shap}}^{\left(3\right)}+\Delta f_{J_{2}}^{\left(3\right)}+\Delta f_{S}^{\left(3\right)}+\Delta f_{1}^{\left(3\right)}+\Delta f_{\text{ion}}^{\left(3\right)}+\Delta f_{\text{tro}}^{\left(3\right)}\right)}$$

where the superscript (2) and (3) denote the respective MWL numbers as shown in Figure 1. Equation 16 can be rewritten as:

(Equation 17) $$\frac{f_{2}^{\prime }}{f_{2}}/\frac{f_{3}^{\prime }}{f_{3}}=\delta {\bar{A}}_{\text{rel}\;}\left(1+\delta f_{\text{dop}\;}+\delta f_{\text{shap}\;}+\delta f_{J_{2}}+\delta f_{S}+\delta f_{1}+\delta f_{\text{ion}\;}+\delta f_{\text{tro}\;}\right)$$

where $\delta f_{i}=\Delta f_{i}^{\left(2\right)}-\Delta f_{i}^{\left(3\right)}$ (where *i* corresponds to dop, shap, *J*_2_,*S*,*l*, ion, and tro in Equation 16). According to the configuration of CSS, the measurement accuracy of the timer is better than 20 ps. Combining with reception/transmission accuracies (20 ps), and a device delay (<30 ps),^33^^,^^49^ results in 2 (3) accuracies of less than 100 ps for *T*_23_,*T*_35_, and*T*_46_. Given that $\delta {\bar{A}}_{\text{rel}}={\bar{A}}_{\text{rel}}^{\left(2\right)}/{\bar{A}}_{\text{rel}}^{\left(3\right)}=1$, and the residual amounts of other frequency shifts are far smaller than the magnitude of ionospheric shifts (10^−13^).^39^^,^^45^ The ratio of two downlinks $\left(\frac{f_{2}^{\prime }}{f_{2}}/\frac{f_{3}^{\prime }}{f_{3}}\right)$ is primarily contain the frequency shift caused by the ionosphere. Using the CSS two downlink frequencies (20.8 GHz and 30.4 GHz) and neglecting third-order ionospheric terms (<10^−20^),^45^ the model is derived by combining Equations 10 and 16 as in^45^:

(Equation 18) $$\frac{f_{2}^{\prime }}{f_{2}}/\frac{f_{3}^{\prime }}{f_{3}}=1+\left(1-\frac{f_{2}^{2}}{f_{3}^{2}}\right)\frac{40.3}{cf_{2}^{2}}\frac{ds}{dt}+\left(1-\frac{f_{2}^{3}}{f_{3}^{3}}\right)\frac{7527}{2f_{2}^{3}}\bar{B_{0}\;\cos\theta }\frac{ds}{dt}$$

Equation 18 indicates that the ionospheric frequency shift can be expressed in terms offrequency *f*_2_. Similarly, this formulation can be extended to the uplink $\frac{f_{1}^{\prime }}{f_{1}}$ and downlink $\frac{f_{2}^{\prime }}{f_{2}}$ to obtain:

(Equation 19) $$\begin{array}{c} \frac{f_{1}^{\prime }}{f_{1}}/\frac{f_{2}^{\prime }}{f_{2}}=\delta {\bar{A}}_{rel}^{\prime }(1+\delta f_{dop}^{\prime }+\delta f_{shap}^{\prime }+\delta f_{J_{2}}^{\prime }+\delta f_{S}^{\prime }+\delta f_{l}^{\prime }+\delta f_{ion}^{\prime }+\delta f_{tro}^{\prime }) \end{array}$$

where $\delta f_{i}^{\prime }=\Delta f_{i}^{\left(1\right)}-\Delta f_{i}^{\left(2\right)}$, *i* has the same meaning as Equation 17, and $\delta {\bar{A}}_{\text{rel}}={\bar{A}}_{\text{rel}}^{\left(1\right)}/{\bar{A}}_{\text{rel}}^{\left(2\right)}$. By combining Equations 10, 18, and 19, we obtain the result given in^45^:

(Equation 20) $$\frac{f_{1}^{\prime }}{f_{1}}/\frac{f_{2}^{\prime }}{f_{2}}=\frac{m_{1}}{m_{2}}\delta {\bar{A}}_{rel}^{\prime }\left(1+\delta f_{dop}^{\prime }+\delta f_{shap}^{\prime }+\delta f_{J_{2}}^{\prime }+\delta f_{S}^{\prime }+\delta f_{1}^{\prime }+\delta f_{tro}^{\prime }\right)$$

where

(Equation 21) $$\begin{array}{c} \frac{m_{1}}{m_{2}}=1+\left(\frac{f_{2}^{2}}{f_{1}^{2}}-1\right)\left(\frac{f_{3}^{2}}{f_{3}^{2}-f_{2}^{2}}\right)\left(\frac{f_{2}^{\prime }}{f_{2}}/\frac{f_{3}^{\prime }}{f_{3}}-1\right)+[\left(\pm \frac{f_{2}^{3}}{f_{1}^{3}}-1\right)-\left(\frac{f_{2}^{2}}{f_{1}^{2}}-1\right)\left(\frac{f_{3}^{2}}{f_{3}^{2}-f_{2}^{2}}\right)\\ \times \left(1-\frac{f_{2}^{3}}{f_{3}^{3}}\right)]\frac{7527}{2f_{2}^{3}}\bar{B_{0}\;\cos\theta }\frac{ds}{dt} \end{array}$$

A comprehensive record of the methodology is provided in Appendix A of Zhang et al. (2023).^45^

However, we actually require the GP of the GS. According to its definition, the GP for ground points at rest relative to the Earth can be expressed as^39^:

(Equation 22) $$W=U+\frac{v^{2}}{2}$$

During microwave signal propagation, the GP of the GS remains constant ($W_{B}=W_{B^{\prime }}$). Furthermore, the change in the gravitational potential due to the CSS’s positional shift is negligible, being less than 0.01 m^2^/s^2^. Therefore, we can approximate $U_{A}=U_{A^{\prime }}$. Substituting Equations 22 and 14 into Equation 20 yields:

(Equation 23) $$\frac{f_{1}^{\prime }}{f_{1}}/\frac{f_{2}^{\prime }}{f_{2}}=\frac{m_{1}}{m_{2}}{\left[1-\frac{1}{c^{2}}\left(W_{B}-U_{A}-\frac{v_{A}^{2}}{2}\right)+\epsilon \left(c^{-4}\right)\right]}^{2}\times \left(1+\delta f_{\text{dop}\;}^{\prime }+\delta f_{\text{shap}\;}^{\prime }+\delta f_{J_{2}}^{\prime }+\delta f_{S}^{\prime }+\delta f_{1}^{\prime }+\delta f_{\text{tro}\;}^{\prime }\right)$$

where $m_{1}/m_{2}$ can be determined by Equation 21, which contained the ionospheric frequency shifts. By using the modified tri-frequency combination model, we can determine the GP of GS.

#### Simulation experiments

The CSS is currently in orbit, but the HPTFS is still under testing, and the space-to-ground OAC comparison data are also not available. To validate the theoretical model proposed in this study, we conducted simulation experiments. The simulation parameters were configured according to the design specifications. The CSS orbit was propagated from the TLEs dataset. For environmental perturbations, the ionospheric frequency shift was calculated using the International Reference Ionosphere (IRI) model,^45^ and the tropospheric frequency shift was computed using data provided by the Vienna mapping function (VMF) data server.^50^^,^^51^ The gravitational potential and GP were derived from the EGM2008 model,^52^ while solid Earth tides were estimated based on the IERS conventions.^48^ Finally, clock data were simulated using a stochastic noise model.^53^^,^^54^

#### Simulation setup

In the simulation experiments, the GS was selected at the Luojia Time and Frequency Geodesy Center (LJTF), whose geographic information parameters are listed in Table 1. The location of LJTF is (30°31′51.90274″ N, 114°21′25.83516″ E) and the ellipsoid height is about 25.728 m. Tthe GP and gravitational potential are 62636467.54 m^2^/s^2^ and 62556081.21 m^2^/s^2^, respectively.

**Table 1:** Details of data processing strategies and options

Parameters	Values
Latitude	30°31′51.90274″ N
Longitude	114°21′25.83516″ E
Ellipsoid Height (h)	25.728 m
Gravitational potential (V)	62556081.21 m^2^*/*s2
Gravity potential (W)	62636467.54 m^2^*/*s2

The simulation was configured to run for 31 days, from 1 to 31 May 2024, with a sampling interval of 1 s. The frequency stabilities of the onboard OAC and the GS OAC were set to 2 × 10^−15^/ $\sqrt{\tau }$ and 1 × 10^−15^/ $\sqrt{\tau }$, respectively. To simulate the received frequencies $f_{1}^{\prime }/f_{1},f_{2}^{\prime }/f_{2}$, and $f_{3}^{\prime }/f_{3}$, the corresponding proper frequencies were defined according to the CSS mission design: an uplink at *f*_1_ = 26.8 GHz (LHCP) and two downlinks at *f*_2_ = 30.4 GHz (RHCP) and *f*_3_ = 20.8 GHz (RHCP).

In the simulation, the coordinates of the LJTF station were assumed to be error-free. The orbital and velocity accuracy of the CSS was set to 5 cm^35^ and 0.1 mm/s,^49^ respectively. Observations commenced once the CSS entered the GS’s field of view, with a cutoff elevation angle of 15° applied to ensure data quality. The frequency shifts induced by the ionosphere and troposphere were simulated using Equations 10 and 11. The employed ionospheric model mitigates approximately 95% of the ionospheric shift,^55^^,^^56^ while the accuracy of zenith troposphere delay (ZTD) is better than 5 cm.^57^^,^^58^ The accuracy of the Earth tide model correction is better than 1 cm.^59^^,^^60^ With these relevant parameters configured, we can begin the simulation process.

#### Generate simulation observations

**Figure 2:**
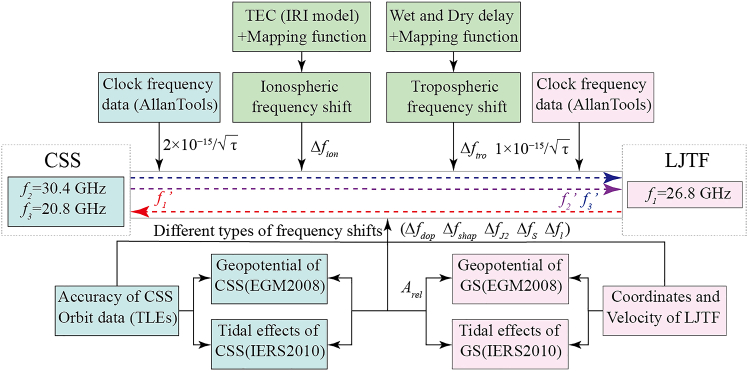
Flowchart of the simulation for one-way microwave frequency observations The colors of the boxes denote the sources of errors: light pink corresponds to related errors of GS, light cyan to those associated with the CSS, and light green to errors arising from atmospheric conditions.

This section details the procedure for generating simulated observational data. Figure 2 illustrates the process of generating simulated frequency observations for one-way MWLs. In our simulation, these observations correspond to the received frequencies of the CSS MWLs ($f_{1}^{\prime }$, $f_{2}^{\prime }$, and $f_{3}^{\prime }$ as Figure 2 shows). For the subsequent calculation of the GP of LJTF, we use the fractional frequencies $f_{1}^{\prime }/f_{1},f_{2}^{\prime }/f_{2},f_{3}^{\prime }/f_{3}$ as the simulation observations.

As established in Section 2.1, various frequency shifts are functions of the relative position and velocity between the CSS and the GS. Therefore, generating simulated observations first requires precise orbital data for the CSS. For this purpose, we utilized the TLEs for the CSS (TIANHE), obtained from Celestrak (https://celestrak.org/NORAD/elements/), to compute the orbital trajectory. The orbital velocity was derived by numerically differentiating the positional data. The resulting orbital elements and velocity serve as reference values. Simulated observational data were then synthesized by adding noise to these references, with the noise levels corresponding to the reported precision of CSS orbit determination (5 cm)^35^ and velocity determination (0.1 mm/s).^49^

With the proper frequencies and orbits, we proceeded to simulate the observational data. A critical component is modeling the clock errors of the OACs, including the transmitter and receiver. These errors are typically characterized in the frequency domain by power-law noise. The power spectral density of the normalized frequency deviation is often modeled as a sum of five independent noise types.^54^ Among them, white frequency modulation (WFM) noise is the dominant contributor, followed by random walk frequency modulation (RWFM) noise. The other three noise types are significantly smaller. The total clock error for an OAC is generated by summing all five noise components.

**Figure 3:**
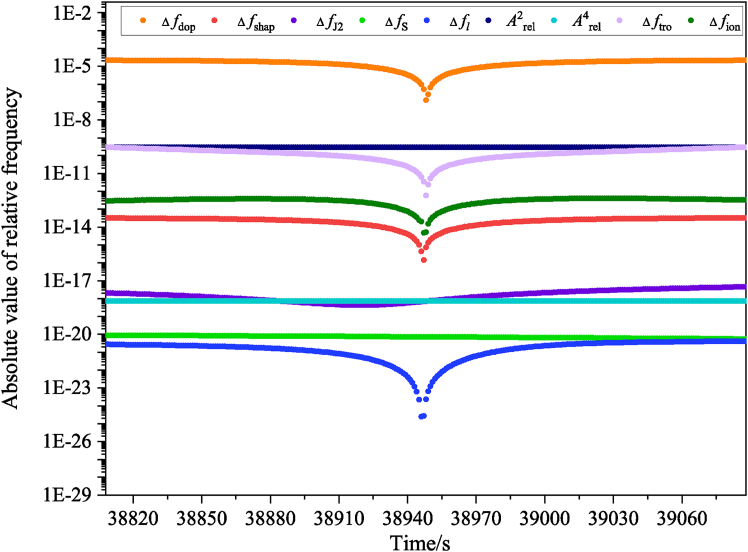
The absolute values of the frequency shifts induced by different error sources during an individual observation

According to Equation 14, precise determination of the relativistic frequency shift requires information not only on the positions and velocities of the CSS and the GS, but also on the gravitational potential and solid Earth tides at their locations. In the data simulation, the gravitational potential at both the CSS and the GS is computed using the EGM2008 model, while solid Earth tide effects are derived from IERS parameters with a sampling interval of 1 s. The dominant contributions to solid Earth tides come from the Moon and the Sun, producing gravitational potential changes of about 4.41 m^2^/s^2^ and 1.60 m^2^/s^2^, respectively.^59^ The resulting variation in gravitational potential at the CSS is estimated to be below 6.27 m^2^/s^2^, and less than 3.16 m^2^/s^2^ at the GS. These variations introduce a relative frequency shift on the order of 10^−17^, which must be corrected using a tide model. The rate of tidal variation at the CSS is about 0.1 m^2^/s^2^, so over short durations (within T_23_ and T_35_), the tidal effect can be treated as constant. The variation rate at the GS is even lower (<0.01 m^2^/s^2^), and the signal propagation time is much shorter than 1 s. Therefore, in the simulation of $f_{1}^{\prime }$, $f_{2}^{\prime }$, and $f_{3}^{\prime }$, tidal influences on both the CSS and the GS are considered constant over a single observation. Figure 3 presents the absolute frequency shifts caused by different error sources in one observation. The relativistic frequency shifts for $A_{\text{rel}}^{2}$ and $A_{\text{rel}}^{4}$ reach magnitudes of 10^−10^ and 10^−19^, respectively.

In addition to relativistic effects, frequency shifts caused by atmospheric propagation, mainly through the ionosphere and troposphere, must also be considered. Based on Equation 10, simulation of the ionospheric frequency shift requires the rate of change of STEC. In this study, zenith TEC derived from the IRI model^61^ is converted to STEC using a mapping function, and the STEC variation rate is obtained by making a difference between two STEC samples.^55^ The term *B*_0_*cos*⁡*θ*, which reflects the influence of the geomagnetic field (modeled via the International Geomagnetic Reference Field, IGRF)^62^ on the MWL signal direction, is used to simulate second-order ionospheric frequency shifts. As shown in Figure 3, the magnitude of these shifts reaches 10^−13^. For the tropospheric contribution, ZTD is taken from the VMF data server (https://vmf.geo.tuwien.ac.at/)^63^ and converted to slant delay using the VMF1 mapping function. The resulting frequency shift, calculated via Equation 11, is on the order of 10^−10^ (Figure 3).

During MWL propagation, the signal is influenced not only by atmospheric conditions but also undergoes various frequency shifts caused by the relative motion between the CSS and GS, as indicated in Equation 4. Consequently, these frequency shifts must be incorporated into the simulation when generating synthetic observations. These shifts include the first-order Doppler shift Δ*f*_dop_, the shift due to the Shapiro effect Δ*f*_shap_, the shift arising from the quadrupole moment $\Delta f_{J_{2}}$, and the shift induced by intrinsic angular momentum Δ*f*_*S*_, among other minor contributions Δ*f*_*l*_. As depicted in Figure 3, the dominant frequency shift stems from the first-order Doppler effect, while the Shapiro effect contributes at a level of approximately 10^−14^, and the remaining three shifts are below 10^−17^. The observed frequencies $f_{1}^{\prime }$, $f_{2}^{\prime }$, and $f_{3}^{\prime }$ are obtained by incorporating the frequency shifts from various error sources into the corresponding proper frequencies *f*_1_,*f*_2_ and *f*_3_.

## Results

Once the simulated observations $f_{1}^{\prime }$, $f_{2}^{\prime }$, and $f_{3}^{\prime }$ are obtained, the GP is derived using the modified tri-frequency combination model presented in this work. The subsequent analysis involves evaluating the accuracy of both the frequency transfer and the GP determination.

### Error analysis of the tri-frequency combination method

**Figure 4:**
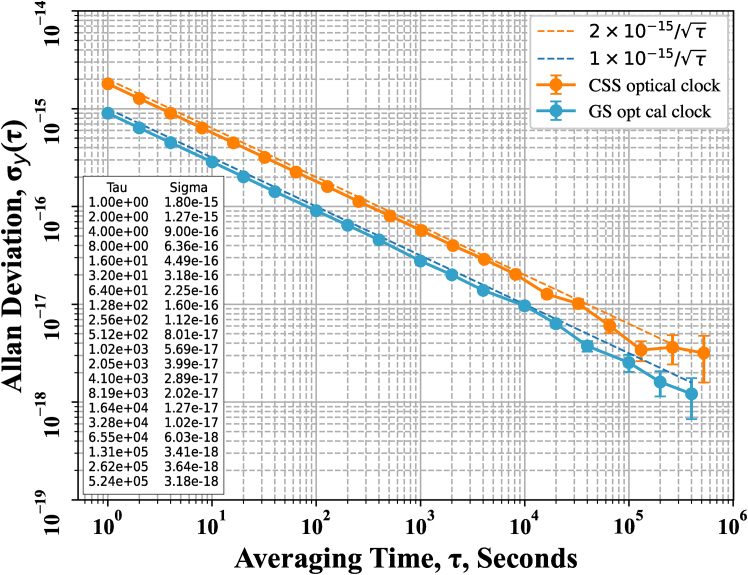
The ADEV distribution of simulated clock data generated by the OACs on CSS and at the LJTF

Before evaluating the frequency transfer results, the performance of the OACs is assessed. The total clock error is synthesized from the five types of clock noise described in Section 3.2. Frequency stability of the OACs is characterized using the Allan deviation (ADEV,.^64^^,^^65^^,^^66^ Figure 4 presents the ADEV distributions for both the CSS and GS OACs. The table in the lower-left corner of Figure 4 lists the ADEV values of the CSS OAC. At 1 s, the frequency stabilities of the CSS and GS OACs are 2 × 10^−15^ and 1 × 10^−15^, respectively. The stability of the CSS OAC reaches 2.89 × 10^−17^ at 4196 s, consistent with its design specifications. For an integration time of 65,536 s, both clocks achieve frequency stability at the 10^−18^ level.

**Figure 5:**
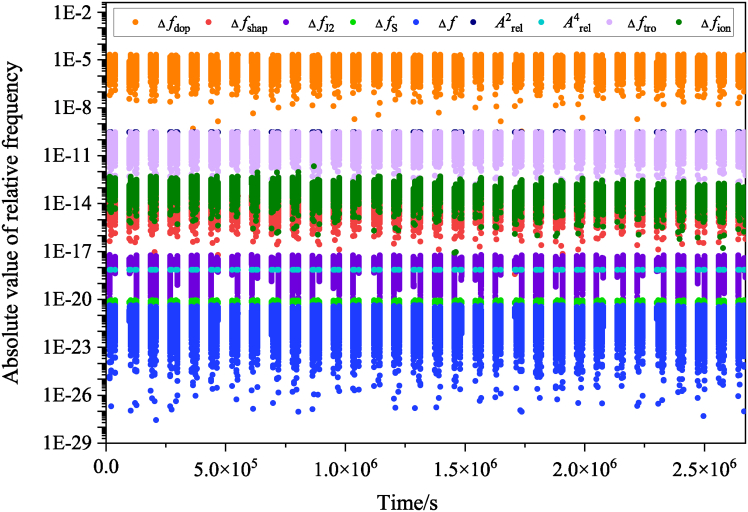
The absolute values of the frequency shifts induced by different error sources over a one-month observation period from May 1 to May 31, 2024

Figure 5 illustrates the magnitudes of frequency shifts in the downlink signal *f*_2_ caused by different error sources over a one-month observation period from May 1 to May 31, 2024. In one-way frequency transfer, the first-order Doppler shift is the dominant error term. Relativistic and tropospheric effects contribute shifts on the order of 10^−10^, while ionospheric and Shapiro effects induce shifts ranging from 10^−15^∼10^−13^. As derived in Section 2.2, the ionospheric frequency shift along the propagation path can be determined using the ratio of the two downlink frequency ratios: $\left(\frac{f_{2}^{\prime }}{f_{2}}/\frac{f_{3}^{\prime }}{f_{3}}\right)$.

The analysis of residuals in the two-downlink ratio $\frac{f_{2}^{\prime }}{f_{2}}/\frac{f_{3}^{\prime }}{f_{3}}$ is based on the similar propagation paths shown in Figure 1, where the signals are transmitted with an interval of T_35_ = 100 ps. Results are presented in Table 2. After removing the relativistic component, the residual first-order Doppler shift in the combined observables is below 5 × 10^−16^, and can be further reduced to the 10^−17^ level via modeling. Although the ratio cancels some common-mode errors, it does not cancel the ionospheric frequency shift because the latter is frequency-dependent. Therefore, the ratio $\frac{f_{2}^{\prime }}{f_{2}}/\frac{f_{3}^{\prime }}{f_{3}}$ cannot eliminate the ionospheric frequency shift. The ionospheric residual thus represents the differential effect between the two frequencies, with a magnitude of 10^−15^*∼* 10^−13^, and is expressed via the ratio $\frac{m_{1}}{m_{2}}$ as a function of frequency *f*_2_.

**Table 2:** The relative magnitudes of the downlink frequency shifts (Δ*f*_*i*_) and residuals of the two-downlink differential (*δf*_*i*_) for each observation

Error Sources	Magnitudes (Δ*f*_*i*_)	Residuals (*δf*_*i*_)
First-order Doppler effect	10^*−*6^*∼* 10^*−*^5	*<* 5*×*10^*−*^16
Shapiro Effect	10^*−*15^*∼* 10^*−*^14	10^*−*25^*∼* 10^*−*^24
Quadrupole Moment (J_2_)	10^*−*18^*∼* 10^*−*^16	10^*−*27^*∼* 10^*−*^26
Intrinsic Angular Momentum (S)	10^*−*20^*∼* 10^*−*^18	*<* 10^*−*^30
Frequency shifts of Δ*f*_*l*_	10^*−*21^*∼* 10^*−*^19	*<* 10^*−*^30
Relativistic Effects	10^*−*11^*∼* 10^*−*^10	–
Tropospheric Effect	10^*−*12^*∼* 10^*−*^10	*<* 10^*−*^20
Ionospheric Effect	10^*−*15^*∼* 10^*−*^13	10^*−*15^*∼* 10^*−*^13

**Figure 6:**
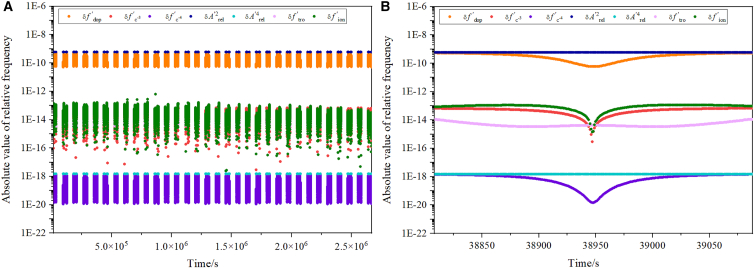
Residuals from different error sources in the ratio of the two downlinks (A) Residuals of different error sources during a one-month observation. (B) Residuals of different error sources during one observation.

Figure 6 presents the residuals of various error sources in the frequency ratio $\frac{f_{1}^{\prime }}{f_{1}}/\frac{f_{2}^{\prime }}{f_{2}}$. Figure 6A corresponds to one month of observations from May 1 to 31, 2024, while Figure 6B displays results from a single randomly selected observation. The combined observation of $f_{1}^{\prime }/f_{1}$ and $f_{2}^{\prime }/f_{2}$ yields first-order Doppler residuals on the order of 10^−10^, which must be corrected using appropriate models. The relativistic frequency shift is twice that of the one-way link, with a magnitude of 10^−10^ (denoted as ${\delta A}_{\text{rel}}^{\prime 2}$ in Figure 6). The ionospheric frequency shift is approximately 10^−13^, and the tropospheric residual ranges from 10^−15^ to 10^−14^. Terms ${\delta f}_{c^{-4}}^{\prime }$ and ${\delta A}_{\text{rel}}^{\prime 4}$ are significantly smaller, at about 10^−19^ to 10^−18^.

**Figure 7:**
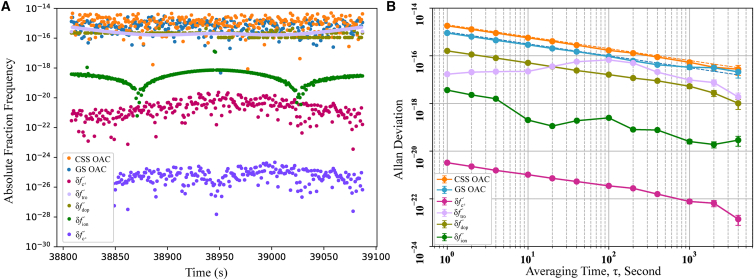
The residuals of different error sources (A) Shows the absolute values of the frequency shift induced by different error sources for a time observation. (B) Is the Allan deviation of different error sources for one month from May 1 to 31, 2024.

To extract the GP difference from the ratio $\frac{f_{1}^{\prime }}{f_{1}}/\frac{f_{2}^{\prime }}{f_{2}}$, the first-order Doppler, ionospheric, tropospheric, and residual Shapiro frequency shifts must be removed. The ionospheric shift is derived from the two-downlink ratio $\frac{f_{2}^{\prime }}{f_{2}}/\frac{f_{3}^{\prime }}{f_{3}}$, while the Doppler, tropospheric, and Shapiro effects are modeled according to the approaches described in Section 2.1. The post-correction residuals are shown in Figure 7. Figure 7A illustrates the residuals for a single observation: The tropospheric and first-order Doppler shifts are below 10^−16^, and the ionospheric and c^−3^ term shifts are below 10^−18^. After model correction, $\delta fc^{-4^{\prime }}$ is reduced to below 10^−25^, and ${\delta A}_{\text{rel}}^{\prime 4}$, which contains the gravitational potential signal, is treated as the estimated value given by Equation 23. Figure 7B shows the Allan deviation of various error sources over one month of observations. All residuals lie below the noise levels of the CSS and GS OACs, indicating that the final accuracy of the GP determination is limited primarily by OAC performance.

### Accuracy analysis of the geopotential

To evaluate the accuracy of the modified tri-frequency combination model, we used the EGM2008 model to calculate the GP of LJTF and treated it as the true value. Based on the analysis of Section 4.1, the residuals of each component in the tri-frequency combined observables have been derived. Using Equation 23, the GP of LJTF can be determined.

As the orbit altitude of the CSS is only about 400 km, when the cutoff elevation angle is set to 15°, the observable duration during each pass of the CSS over the GS is only about 300 s. The average value and uncertainty calculated from the 300 s data are taken as the GP value and uncertainty (*σ*_*i*_, *i*is the number of observation times) for one observation. Since each observation segment is discontinuous, when calculating the monthly GP value, the weight is assigned based on the *σ*_*i*_ of each observation. For one-month observations, the mean GP can be calculated by $W_{mean}=\sum_{1}^{n}\;w_{i}/\sum_{1}^{n}\;w_{i}W_{i}$, where *i*is the number of observation times, *w*_*i*_ and *W*_*i*_ are the weight and GP for the *i-th* observation. The weighted mean standard deviation (STD) formula is $\sigma_{w}=\sqrt{\sum_{1}^{n}\;\delta_{i}^{2}w_{i}^{2}}/\sum_{1}^{n}\;w_{i}$, which was used to evaluate the one-month data. By the weighted average method, the mean value and the accuracy of the GP over one month can be calculated.^38^

**Figure 8:**
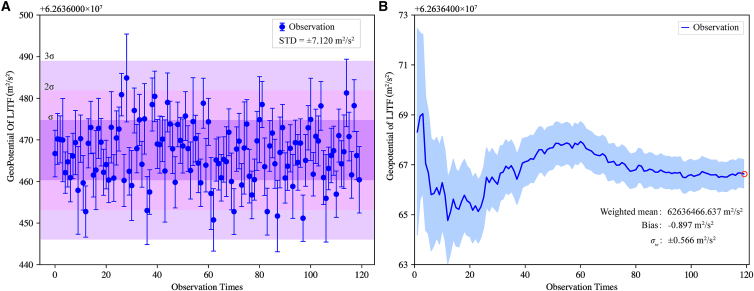
The GP at LJTF was determined using the modified tri-frequency combination method for the entire month of May 2024 (A) GP values derived from 120 individual observations (blue circles), with pink bands indicating the σ, 2σ, and 3σ ranges, respectively. (B) Cumulative weighted average and STD of the GP values.

Figure 8 displays the monthly averaged GP results for the LJTF site in May 2024, obtained via the modified tri-frequency combination model. The error bar chart is plotted based on the mean value and uncertainty of each observation, in Figure 8A the points represent the mean values of GP, and the error bars indicate the uncertainty of each point. A total of 120 observations were acquired in May 2024. Statistical analysis of these data yields a STD of 7.12 m^2^/s^2^ for a single observation. All results fall within the 3σ bound, and five values lie outside the 2 range, accounting for 6.67% of the dataset. The weighted average and its uncertainty were then computed based on the accuracy of each measurement (Figure 8B). The weighted mean stabilizes after approximately 80 observations. The final result, based on all 120 observations, is (62636466.637 ± 0.566) m^2^/s^2^. Compared to the true GP value at LJTF, the bias is approximately 0.897 m^2^/s^2^.

To further validate the modified tri-frequency combination model, additional experiments were conducted. The weighted mean values and STDs of the GP at the LJTF site, calculated for the period from April to September 2024, are summarized in Table 3. The weighted mean GP values of LJTF from April to September 2024 are 62636466.867 m^2^/s^2^, 62636466.637 m^2^/s^2^, 62636466.898 m^2^/s^2^, 62636468.231 m^2^/s^2^, 62636468.362 m^2^/s^2^, 62636466.599 m^2^/s^2^, respectively. The absolute biases for all six months are below 1 m^2^/s^2^, and the STDs are under 0.7 m^2^/s^2^, corresponding to a height equivalent of about 7 cm.

**Table 3:** The GP results and their accuracy for the LJTF calculated by the modified tri-frequency combination model

Month	GP (m^2^/s^2^)	Bias (m^2^/s^2^)	STD (m^2^/s^2^)
Apr.	62636466.867	−0.667	±0.631
May	62636466.637	−0.897	±0.566
Jun.	62636466.898	−0.636	±0.649
Jul.	62636468.231	0.697	±0.626
Aug.	62636468.362	0.828	±0.623
Sep.	62636466.599	−0.935	±0.634

Our simulation validates the capability of the modified tri-frequency combination method to effectively eliminate various systematic errors, extract the gravitational frequency shift effect, and ultimately determine the GP difference, achieving a determination accuracy equivalent to a centimeter-level height discrepancy.

## Discussion

According to the characteristics of the MWLs onboard the CSS, this study modified the tri-frequency combination model for GP determination by extending it to include terms up to the c^−4^ order. The model systematically incorporates the impacts of clock noise, ionospheric and tropospheric delays, and tidal effects on MWL frequency signals, with corresponding error correction models established accordingly. Analysis indicates that the tropospheric frequency shift is on the order of 10^−10^, while the ionospheric shift is approximately 10^−13^. By combining two downlink signals, non-ionospheric errors are effectively eliminated, and the residual of first-order Doppler shift is about 2∼3 orders smaller than the first-order ionospheric frequency shift. As a result, the ionospheric shift can be expressed as a quantity related to the downlink frequency *f*_2_. Through the combination of the uplink frequency *f*_1_ and one downlink frequency *f*_2_, the gravitational frequency shift is doubled, whereas first-order Doppler, tropospheric, and other systematic errors are suppressed. The first-order ionospheric frequency shift is derived from the ratio of two downlink combinations. After processing with the modified tri-frequency combination model, all residual errors are smaller than the noise level of the OACs. Therefore, in practice, the stability of OACs ultimately limits the precision of GP difference measurements.

The modified tri-frequency combination model proposed in this work allows for the determination of GP at arbitrary GSs. Based on the designed accuracy of OACs aboard the CSS and at GSs, as well as models of various error sources, we constructed simulated observation values and conducted a six-month simulation experiment. The results of the simulation experiments showed that the GP biases at the LJTF station are below 1 m^2^/s^2^ for all six months, with STDs under 0.7 m^2^/s^2^. This indicates that one month of accumulated observations is sufficient to determine the GP with an accuracy corresponding to better than 7 cm in height. The simulations also confirm that the modified model achieves frequency transfer accuracy at the c^−4^ order, meeting the requirement for frequency comparisons below 10^−18^. By utilizing the combination of three MWLs from the CSS, the first-order ionospheric frequency shift, first-order Doppler shift, and tropospheric frequency shift can be effectively eliminated. This allows precise extraction of the gravitational frequency shift signal, ultimately enabling high-precision determination of GP.

## Resource availability

### Lead contact

Further information and requests for resources should be directed to the lead contact, Chenxiang Wang, wangchenxiang@whu.edu.cn.

### Materials availability

This study did not generate new unique materials.

### Data and code availability

In this study, the basic data of the Earth orientation parameter data were downloaded from the IERS website (https://datacenter.iers.org/data/latestVersion/223_EOP_C04_14.62-NOW.IAU1980223.txt). TLEs data can be obtained from https://celestrak.org/NORAD/elements/stations.txt. The parameters of the troposphere can be downloaded from the website of VMF Data Server (https://vmf.geo.tuwien.ac.at/trop_products/GRID/1x1/VMF3/VMF3_OP/). The code supporting the current study has not been deposited in a public repository because the funding supporting this manuscript has not been completed yet, but it is available from the corresponding author on request.

### Limitations of the study

Although atomic clocks are already in operation in space—onboard both the CSS and the ACES mission, no space-to-ground time and frequency comparison data based on OACs have been acquired to date. Therefore, it is difficult to conduct practical measurement experiments in the short term. Nonetheless, with the ongoing progress of space-based atomic clock initiatives, real observational data are anticipated to become available in the future. In that context, this study will offer essential theoretical support for the analysis of such experimental data.

## Acknowledgments

This study is supported by the 10.13039/501100011354State Key Laboratory of Geo-Information Engineering, the Key Laboratory of Surveying and Mapping Science and Geospatial Information Technology of 10.13039/100015809MNR, 10.13039/100022811CASM (2024-01-01), the Open Found of Hubei Luojia Laboratory (no. 250100013), 10.13039/501100002858China Postdoctoral Science Foundation (Certificate Number: 2024M752480), the Henan Province Major Science and Technology Special Project (no. 251100240100), the 10.13039/501100001809National Natural Science Foundation of China (grant nos. 42388102 and 42304095), and the State Key Laboratory of Spatial Datum (grant no. SKLSD2025-ZZ-04).

## Author contributions

Conceptualization: P.Z. and C.W.; methodology: P.Z., C.W., and W.X.; software: P.Z.; data curation: P.Z., C.W.; formal analysis: P.Z., C.W.; visualization: P.Z. and C.W.; writing – original draft: P.Z. and C.W.; writing - review and editing: P.Z., C.W., and W.X.; funding acquisition: P.Z., W.X., and C.W.; supervision: C.W.

## Declaration of interests

The authors declare no competing interests.

## STAR★Methods

### Key resources table

**Table:** 

REAGENT or RESOURCE	SOURCE	IDENTIFIER
**Deposited data**		

EOP Data	IERS	https://datacenter.iers.org/data/latestVersion/
TLEs Data	NORAD	https://celestrak.org/NORAD/elements/stations.txt
Tropospheric data	Vienna Mapping Functions Data Server	https://vmf.geo.tuwien.ac.at/trop_products/GRID/1x1/VMF3/VMF3_OP/

**Software and algorithms**		

Python3.9	JetBrains	https://www.python.org/downloads/

### Method details

#### Model

Firstly, we derive the one-way frequency transfer model:

(Equation 24) $$\frac{\nu_{B}}{\nu_{A}}={\bar{A}}_{\text{rel}\;}\left(1+\Delta f_{\text{dop}\;}+\Delta f_{\text{shap}\;}+\Delta f_{J_{2}}+\Delta f_{S}+\Delta f_{l}+\Delta f_{\text{ion}\;}+\Delta f_{\text{tro}\;}\right)$$

Then, we actually require the GP of the GS. According to its definition, the GP for ground points at rest relative to the Earth can be expressed as^39^:

(Equation 25) $$W=U+\frac{v^{2}}{2}$$

During microwave signal propagation, the GP of the GS remains constant ($W_{B}=W_{B^{\prime }}$). Furthermore, the change in the gravitational potential due to the CSS’s positional shift is negligible, being less than 0.01 m^2^/s^2^. Therefore, we can approximate $U_{A}=U_{A^{\prime }}$. $\frac{f_{1}^{\prime }}{f_{1}}/\frac{f_{2}^{\prime }}{f_{2}}$ can be expressed as:

(Equation 26) $$\frac{f_{1}^{\prime }}{f_{1}}/\frac{f_{2}^{\prime }}{f_{2}}=\frac{m_{1}}{m_{2}}{\left[1-\frac{1}{c^{2}}\left(W_{B}-U_{A}-\frac{v_{A}^{2}}{2}\right)+\epsilon \left(c^{-4}\right)\right]}^{2}\times \left(1+\delta f_{\text{dop}\;}^{\prime }+\delta f_{\text{shap}\;}^{\prime }+\delta f_{J_{2}}^{\prime }+\delta f_{S}^{\prime }+\delta f_{1}^{\prime }+\delta f_{\text{tro}\;}^{\prime }\right)$$

where $m_{1}/m_{2}$ can be determined by Equation 21, which contained the ionospheric frequency shifts. By using the modified tri-frequency combination model, we can determine the GP of GSs.

#### Data and preprocessing

The simulation parameters were configured according to the design specifications. The CSS orbit was propagated from the TLEs dataset. For environmental perturbations, the ionospheric frequency shift was calculated using the International Reference Ionosphere (IRI) model,^45^ and the tropospheric frequency shift was computed using data provided by the Vienna Mapping Functions (VMF) data server.^50^^,^^51^ The gravitational potential and GP were derived from the EGM2008 model,^52^ while solid Earth tides were estimated based on the IERS conventions.^48^ Finally, clock data were simulated using a stochastic noise model.^53^^,^^54^

In the simulation experiments, the GS was selected at the Luojia Time and Frequency Geodesy Center (LJTF), whose geographic information parameters are listed in Table 1. The location of LJTF is (30°31′51.90274″ N, 114°21′25.83516″ E), and the ellipsoid height is about 25.728 m. The GP and gravitational potential are 62636467.54 m^2^/s^2^ and 62556081.21 m^2^/s^2^, respectively. The simulation was configured to run for 31 days, from 1 to 31 May 2024, with a sampling interval of 1 s. The frequency stabilities of the onboard OAC and GS OAC were set to 2 × 10^−15^/ $\sqrt{\tau }$ and 1 × 10^−15^/ $\sqrt{\tau }$, respectively. To simulate the received frequencies $f_{1}^{\prime }/f_{1},f_{2}^{\prime }/f_{2}$, and $f_{3}^{\prime }/f_{3}$, the corresponding proper frequencies were defined according to the CSS mission design: an uplink at *f*_1_ = 26.8 GHz (LHCP) and two downlinks at *f*_2_ = 30.4 GHz (RHCP) and *f*_3_ = 20.8 GHz (RHCP). The coordinates of the LJTF station were assumed to be error-free. The orbital and velocity accuracy of the CSS was set to 5 cm^35^ and 0.1 mm/s,^49^ respectively. Observations commenced once the CSS entered the GS’s field of view, with a cutoff elevation angle of 15° applied to ensure data quality. The frequency shifts induced by the ionosphere and troposphere were simulated using (Equation 10), (Equation 11). The employed ionospheric model mitigates approximately 95% of the ionospheric shift, while the accuracy of zenith troposphere delay (ZTD) is better than 5 cm.^57^^,^^58^ The accuracy of the Earth tide model correction is better than 1 cm.^59^^,^^60^

For one month observations, the mean GP can be calculated by $W_{mean}=\sum_{1}^{n}\;\frac{w_{i}}{\sum_{1}^{n}\;w_{i}}W_{i}$, where *i*is the number of observation times, *w*_*i*_ and *W*_*i*_are the weight and GP for the *i-th* observation. The weighted mean standard deviation formula is $\sigma_{w}=\sqrt{\sum_{1}^{n}\;\delta_{i}^{2}w_{i}^{2}}/\sum_{1}^{n}\;w_{i}$, which is used to evaluate the one-month data. By the weighted average method, the mean value and the accuracy of the GP over one month can be calculated.^38^

#### Data analysis

The results of the simulation experiments showed that the GP biases at the LJTF station are below 1 m^2^/s^2^ for all six months, with STDs under 0.7 m^2^/s^2^. This indicates that one month of accumulated observations is sufficient to determine the GP with an accuracy corresponding to better than 7 cm in height. The simulations also confirm that the modified model achieves frequency transfer accuracy at the c^−4^ order, meeting the requirement for frequency comparisons below 10^−18^. By utilizing the combination of three MWLs from the CSS, the first-order ionospheric frequency shift, first-order Doppler shift, and tropospheric frequency shift can be effectively eliminated. This allows precise extraction of the gravitational frequency shift signal, ultimately enabling high-precision determination of GP.

### Quantification and statistical analysis

This study does not include statistical analysis or quantification.

### Additional resources

This study has not generated or contributed to a new website/forum and it is not part of a clinical trial.
